# Autoregulation of Woven Fabric Structure: Image-Based and Regression Analysis of Structural Homogeneity Under Varying Weaving Parameters

**DOI:** 10.3390/ma18153554

**Published:** 2025-07-29

**Authors:** Magdalena Owczarek

**Affiliations:** Institute of Architecture of Textiles, Faculty of Textiles and Design, Lodz University of Technology, 90-543 Lodz, Poland; magdalena.owczarek@p.lodz.pl

**Keywords:** weaving process parameters, structural homogeneity, fabric autoregulation, plain weave, twill weave, warp grouping, image analysis, inter-thread pores, filtration textiles, composite reinforcement

## Abstract

This study investigates the influence of weaving process parameters on the structural homogeneity of woven fabrics, with a focus on the structural autoregulation phenomenon. Two experimental fabric groups of 30 each, plain and twill weaves, were produced using varied loom settings: shed closure timing, lease rod position, backrest roller position, warp pre-tension, and yarn twist direction. Structural uniformity was assessed using a proprietary method and the MagFABRIC 2.1. image analysis system, which quantify intra-repeat, inter-repeat, and global inhomogeneity. This method uses the size, shape, and location of inter-thread pores as well as warp and weft pitches. The results indicate that autoregulation can reduce local structural disturbances, including warp yarn grouping. In plain weaves, loom parameters and humidity significantly contributed to structural autoregulation. In contrast, twill weaves demonstrated dominant internal feedback mechanisms, significantly influenced by yarn twist direction. Regression models at F = 10 revealed nonlinear interactions, confirming autoregulation and experimentally supporting Nosek’s quasi-dynamic theory for these types of fabrics. The results of these studies have practical relevance in high-performance textiles such as filtration, barrier fabrics, and composite reinforcements, where local structural deviations critically affect the functional properties of fabrics.

## 1. Introduction

The homogeneity of a woven fabric structure plays a critical role in determining its physical and mechanical properties. It is particularly important in the context of filtration and barrier functions, air permeability, and mechanical strength, especially in technical and high-performance applications. Numerous studies have investigated the influence of fabric and yarn parameters such as weave type, density, and finishing on air permeability [1,2,3,4,5]. For protective fabrics (e.g., medical or filtration textiles), pore uniformity in fabric structure is essential to ensure consistent protection against microorganisms, solid particles, or UV radiation [6,7,8,9,10]. Fabric mechanical characteristics, such as tensile, tear, and bending strength [11,12,13], or deformation behavior in composites [14,15], can be affected by structural irregularities (e.g., variability of yarn alignment).

High fabric homogeneity depends on the weaving process parameters. The ability of woven structures to respond dynamically to disturbances introduced by the loom, referred to as structural autoregulation, remains under-researched. Loom parameters, such as shed closure timing and initial warp tension, significantly influence structural changes in woven fabrics, affecting attributes including thickness, weft density, fabric cross-section, and mechanical properties (breaking force, elongation at break, static friction force, and static friction coefficient) [16]. Increased warp tension can reduce tear strength, tensile strength, and breakage strength, particularly in the warp direction [17].

Adjustments to the backrest and dropper positions, as well as their height, influence the warp tension and final fabric structure. Moving the backrest backward reduces the tension, while increasing its height raises the tension. The required tension also depends on the weave pattern; for example, plain weaves require higher tension than other types [18]. The timing of shed closure affects weaving resistance and fabric density; optimal settings enable the production of denser fabrics [19]. A low shedding angle and high take-up speed help maintain uniform tension, which is particularly important for stiff, non-stretchable yarns [20].

The loom type and setting can cause local variations in warp and weft tension, impacting properties such as tensile strength, bending, shearing, and surface characteristics [21]. Fabrics woven on different rapier looms may have different extensibility and bending rigidity, even within the same loom width. Higher warp tension is often observed in the central area of the loom, resulting in greater weft crimp and a smoother fabric surface compared to the edges [22]. Irregular warp tension across the fabric width contributes to structural inhomogeneity. Key weaving parameters that influence homogeneity include the friction coefficient and weft pretension. Increasing these factors improves warp tension uniformity and reduces fluctuations [23].

Structural changes in fabrics result from the complex interplay of weaving parameters reflecting the inherent autoregulatory behavior of the textile system. The concept of structural autoregulation was first introduced by the Czech researcher Nosek [24]. According to Nosek’s theory, the loom, warp, and fabric are considered as a system in which signals propagate. This theory describes the ability of woven structures to compensate for disturbances through a system of internal and external feedback loops. The external feedback includes mechanical elements (e.g., the reed or tensioning devices), while internal feedback refers to the spontaneous ability of the structure to restore equilibrium in subsequent weaving cycles. Nosek’s theory was later extended by Masajtis [25], who further developed the concept.

Despite these fundamental contributions, current research lacks experimental validation of such feedback-driven behaviors under real weaving conditions. Furthermore, it is still unclear how environmental factors (e.g., humidity, temperature) and yarn construction parameters (e.g., twist direction) affect the dynamics of structural autoregulation. Understanding this mechanism is crucial for analyzing structural defects, optimizing weaving parameters, and designing advanced woven materials with predictable uniformity and repeatable mechanical properties. This study hypothesizes that specific combinations of weaving parameters, humidity and temperature conditions, and yarn properties can induce structural autoregulation, observed as a measurable improvement in fabric structure uniformity under actual weaving process conditions for two different fabric weaves and different looms.

This paper aims to investigate the influence of weaving parameters, loom settings, and yarn characteristics on the autoregulatory behavior of woven structures in plain and twill fabrics. Structural inhomogeneity was evaluated using image analysis, based on a previously developed method and MagFABRIC 2.1. software (authored by the researcher and used at Lodz University of Technology, Lodz, Poland) [26,27,28,29]. A critical aspect of morphometric image analysis is the accurate execution of image acquisition, preprocessing, and thresholding algorithms. Key indicators of structural variability—such as the size, shape, and location of inter-thread pores (ITPs), the warp and weft pitches, and intra-repeat (IAR) and inter-repeat (IER) inhomogeneity within weave patterns—provide valuable insight into autoregulation and uniformity within and between pattern repeats.

Recent studies have shown that image analysis and machine learning can effectively predict porosity also in knitted fabrics, confirming the potential of digital tools for textile structure evaluation [30].

## 2. Materials and Methods

### 2.1. Yarns Used in the Experimental Fabrics

**Figure 1:**
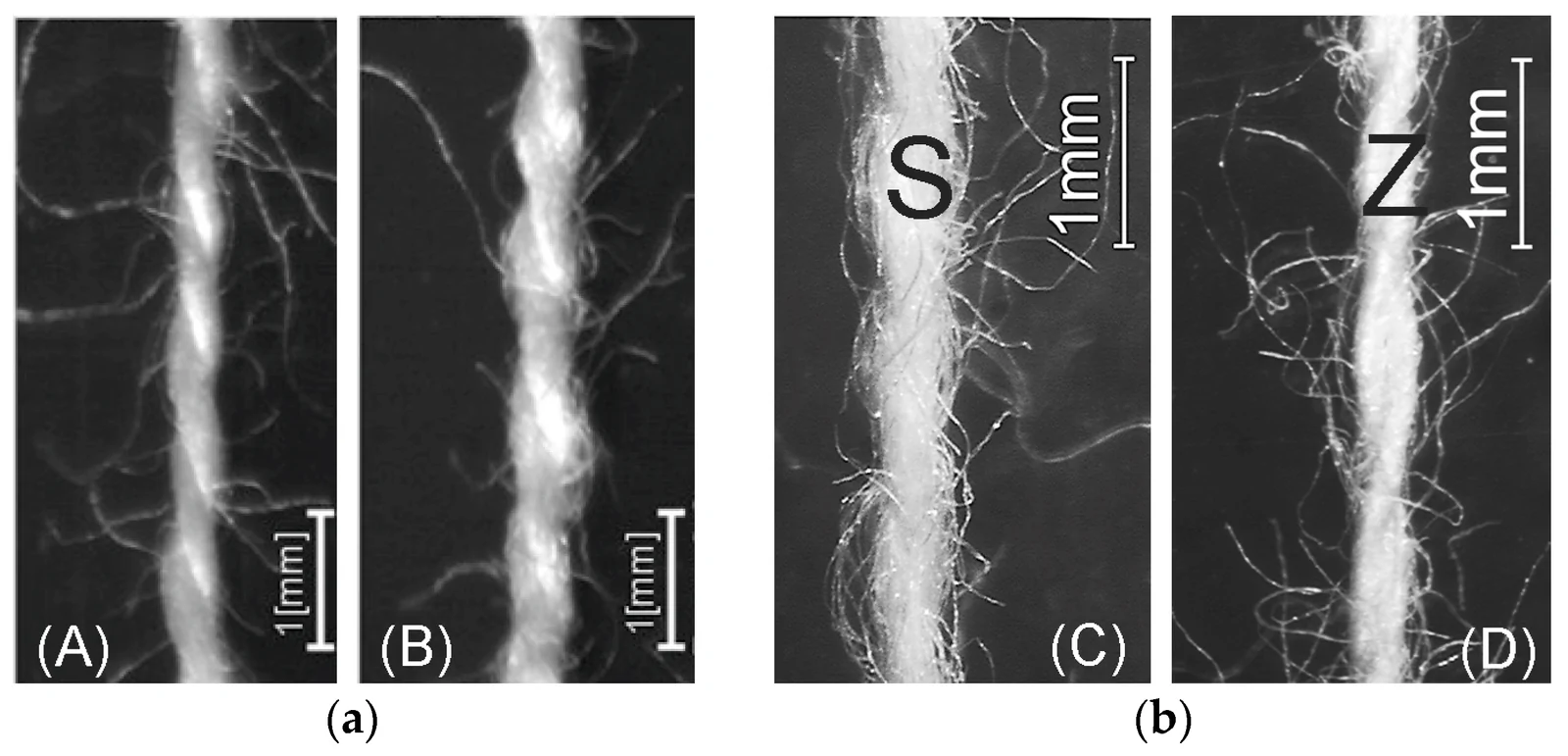
Double yarns made of 100% cotton twin ring-spun yarns (20 × 2 Tex) produced on a ring spinning frame: (**a**) warp (A) and weft (B) used in plain fabrics; (**b**) warp (C) and/or weft (D) used in twill fabrics.

The analysis was carried out on two experimental fabrics—plain and twill weaves—and on different weaving machines. The fabrics were made from 20 × 2 [Tex] cotton yarns, as shown in Figure 1. In the plain fabrics, the warp and weft were made from two separate yarns, (A) and (B), respectively, (Figure 1a). In twill weave fabrics, the warp and weft yarns varied according to the experimental plan. The warp beam was threaded in three sections: one with S-twist warp yarns (C), one with alternating S- and Z-twist warp yarns (C and D), and one with Z-twist warp yarns (D), (Figure 1b).

Cotton was selected due to its high sensitivity to weaving parameters, the natural variability in yarn structure, and its widespread use in both apparel and technical textiles.

The measured parameters of the yarns used in the experiments are presented in Table 1. Yarn characteristics were assessed using the USTER^®^ TESTER system (Uster Technologies AG, Uster, Switzerland) and verified against USTER^®^ STATISTICS 2018. According to global reference data (50% level in USTER Statistics 2018), yarns A and D exhibit good breaking force and CVm% values. Warp yarn A demonstrates high tenacity but low breaking elongation, which is typical for warp applications, whereas warp yarn D has lower tenacity (cN/tex).

The weft yarn B contains a high number of thin places (60) and neps (445), which may affect the uniformity of the fabric structure. In contrast, weft yarn C (used in twill fabrics) exhibits exceptionally high breaking elongation (12.88%), which is typical and may indicate excessive looseness. Other parameters, such as CVm% and related values, remain within acceptable limits.

Yarn hairiness generally falls at or slightly above the global average (50% level in USTER Statistics 2018). However, yarn C shows significantly elevated hairiness (8.06), which could impact the visual uniformity of the fabric structure, particularly in terms of ITPs. Hairiness was measured using the USTER^®^ TESTER system, which quantifies the total length of protruding fibers per unit of yarn length according to standard USTER methodology.

### 2.2. Fabric Weaving Parameters

This study analyzed two groups of experimental fabrics of 30 each: plain and twill weave. Plain and twill weaves were selected as representative fabric architectures, characterized by fundamentally different interlacing patterns and structural organization, which influence their predisposition to autoregulation. Structural variation was introduced by adjusting the weaving process parameters on two different mechanical looms under controlled conditions. This approach enabled a comprehensive investigation of the autoregulation phenomenon.

Since the SAURER W100 loom (Saurer AG, Arbon, Switzerland) offers a wide range of adjustable weaving parameters, it was selected for the plain weave study to enable a detailed analysis of process-driven structural variation. However, due to design limitations allowing only plain weave production, the SACM-MAV180 loom (SACM, Société Alsacienne de Constructions Mécaniques, Mulhouse, France) was used for the 1/4 Z twill fabrics. This allowed the application of twill weave and the introduction of yarn twist direction as an additional process variable, extending the scope of autoregulation analysis to a different fabric architecture.

In the first stage, a SAURER W100 loom was selected due to its extensive range of adjustable process parameters, allowing for a detailed analysis of autoregulation effects. The yarn parameters (Table 1), plain weave structure, warp density (230 ends/dm), and weft density (130 picks/dm) were kept constant. The following weaving parameters were varied for the 30 plain weave fabrics (Table 2):

- Xp_1_ Shed closure timing (388.8–331.2°): open, closed, or crossed shed,
- Xp_2_ Lease rod position (43–73 cm) from the geometric center of the harness,
- Xp_3_ Backrest roller position (82–90 cm) from the center position,
- Xp_4_ Warp pre-tension (5.93–31.91 cN/thread).

In the second stage, the focus was placed on assessing the impact of yarn twist direction on autoregulation. The 30 twill weave fabrics were woven on a SACM-MAV180 laboratory loom, with variations in the following parameters (Table 2):

- Xt_1_ Backrest roller position (100–108 cm) from the center position,
- Xt_2_ Shed closure timing (360.0–303.0°): closed or crossed shed,
- Xt_3_ Warp yarn twist direction (S/S, S/Z, Z/Z),
- Xt_4_ Weft yarn twist direction (S/S, S/Z, Z/Z).

**Table 2:** Parameters of plain and twill fabrics used in the experimental design PS/DS-P:λ(λ), including actual values corresponding to normalized level ${\hat{x}}_{k}$ ∈ (−α, −1, 0, 1, + α), where α = 2.

Fabric	Parameter x_k_	Range (x_kmin_–x_kmax_)	Actual Value for Coded Level (−2, −1, 0, 1, + 2)				
**Plain**	Xp_1_ Shed closure timing	388.8–331.2°	388.8	374.4	360	345.6	331.2
	Xp_2_ Lease rod position	95–125 cm	95	102.5	110	117.5	125
	Xp_3_ Backrest roller position	82–90 cm	82	84	86	88	90
	Xp_4_ Warp pre-tension	5.93–31.91 cN/thread	5.93	12.76	19.15	25.53	31.91
**Twill**	Xt_1_ Backrest roller position	100–108 cm	100	102	104	106	108
	Xt_2_ Shed closure timing	360.0–303.0°	360	330	320	310	303
	Xt_3_ Warp yarn twist direction	S, S/Z, Z	S/S	S/S	S/Z	Z/Z	Z/Z
	Xt_4_ Weft yarn twist direction	S, S/Z, Z	S/S	S/S	S/Z	Z/Z	Z/Z

Note: Normalized levels represent coded values used in the experimental matrix: ${\hat{x}}_{k}$ = −2: minimum value of the variable, ${\hat{x}}_{k}$ = −1: lower intermediate value, ${\hat{x}}_{k}$ = 0: central value (range midpoint), ${\hat{x}}_{k}$ = +1: upper intermediate value, ${\hat{x}}_{k}$ = +2: maximum value of the variable.

**Figure 2:**
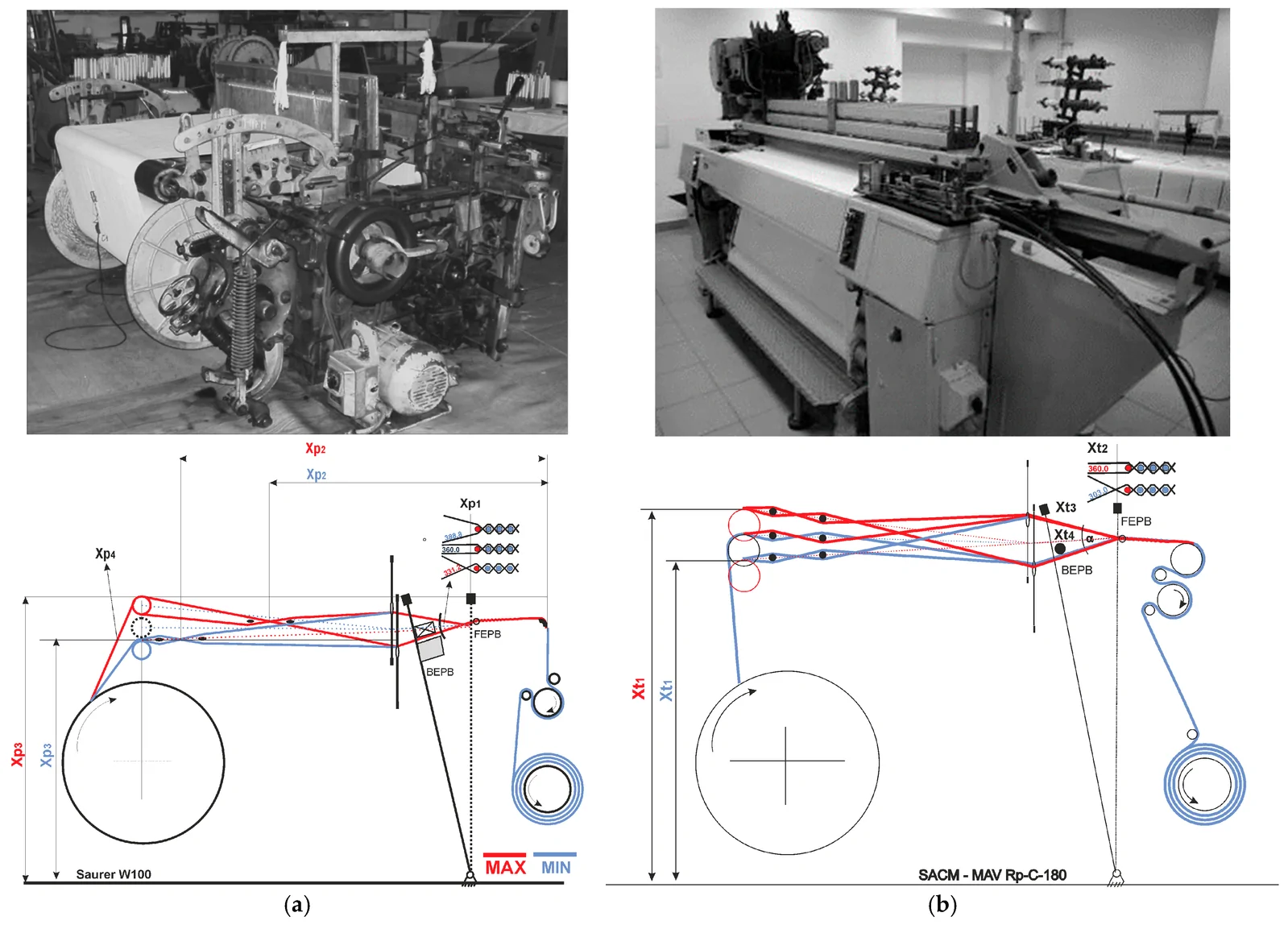
Visualization of weaving machines used in experiments and process parameters on a loom for (**a**) SAURER W100 loom, plain weave fabrics Xp_1_–Xp_4_, and (**b**) SACM-MAV180 loom, twill weave fabrics Xt_1_–Xt_4_, where FEPB—the front extreme position of the beater, and BEPB—the back extreme position of the beater.

For the twill weave fabrics, the yarn parameters (Table 1), warp density (230 ends/dm), weft density (210 picks/dm), warp pre-tension (40 cN/thread), and lease rod position (50 cm from the geometric center of the harness) were kept constant. Images of looms and schematic visualization of the weaving process parameters positions on the loom are shown in Figure 2.

### 2.3. Experimental Design for Fabric Production

To design and produce two groups of fabrics—plain and twill weave—for the study, various experimental designs were analyzed. Ultimately, the polyselective, rotational-uniform PS/DS-P: λ(λ) design was selected, incorporating four input variables at five levels each. The normalization of input variable values was performed using the following equation:

(1) $$x_{kn}={\bar{x}}_{k}+\frac{{\bar{\hat{x}}}_{k}\left(x_{kmax}-x_{kmin}\right)}{2\alpha }$$

where

- *α* = 2—tabulated value,
- *x_kmax_*—maximum value in the range,
- *x_kmin_*—minimum value in the range,
-
${\bar{x}}_{k}$ —the midpoint of the range,
-
${\bar{\hat{x}}}_{k}$ —normalized value (coded level),
-
$x_{kn}$ —target (actual) value.

The experimental design matrix consisted of

- Core design points: n_k_ = 2^i^ = 2^4^ = 16,
- Star points: n_α_ = 2i = 2 × 4 = 8,
- Center points: n_0_ > 1; n_0_ = 7,
- Total number of trials: n = n_k_ + n_α_ + n_0_ = 31.

**Figure 3:**
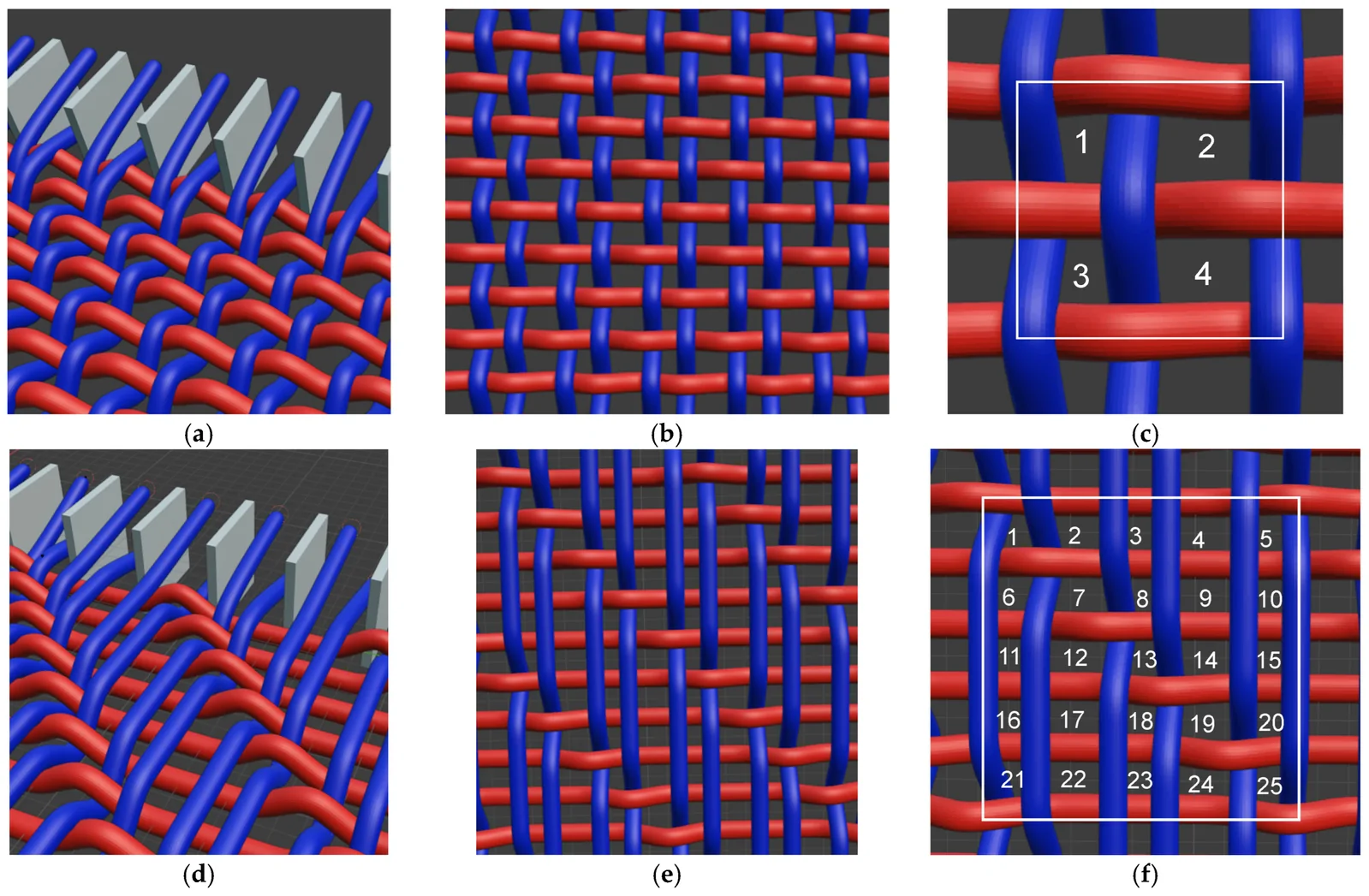
Visualization of the effect of double warp threading through the reed: (**a**) model of fabric formation on the loom in plain weave; (**b**) grouping of warp yarns in plain weave; (**c**) weave repeat in plain fabric with four ITPs; (**d**) model of fabric formation on the loom in twill weave; (**e**) grouping of warp yarns in twill weave; (**f**) weave repeat in twill fabric with twenty-five ITPs.

The warp density for both fabric groups is 230 ends/dm, which represents an average value determined by the reed construction and threading method. Threading two warp yarns per dent increases the fabric density. However, if weaving parameters are not properly adjusted, this setup may cause warp yarn to group within the reed dents, resulting in irregular spacing and ITPs, as illustrated in Figure 3 for a plain weave example. When optimal weaving parameters are applied, the phenomenon of structural autoregulation can be observed. This effect manifests as the equalization of warp spacing and ITP size, leading to a more homogeneous fabric structure.

### 2.4. Procedure for Sample Selection, Image Acquisition, Preprocessing, and ITP Identification

The fabric samples were conditioned under standard laboratory conditions (relative humidity: 65 ± 4%, temperature: 20 ± 2 °C) by ISO 139:2005 [31]. For each fabric, 30 images were captured diagonally across the width using an MST Zoom 1302 CB stereomicroscope (PZO, Warsaw, Poland), a CCD-4012A VideoTronic camera (VideoTronic GmbH, Berlin, Germany), and an Olympus Highlight 3100 transmitted light source (Olympus Corporation, Tokyo, Japan). Imaging was performed at 1.25× magnification, covering an area of 3 × 3 mm^2^ per image. This corresponds to approximately 8 × 5 threads with 28–36 ITPs in plain weave fabrics and 8 × 7 threads with 36–49 ITPs in twill weave fabrics, ensuring high accuracy in ITP identification. Images were acquired at a resolution of 1024 × 1024 pixels with a grayscale depth of 8 bits (0–255 range).

**Figure 4:**
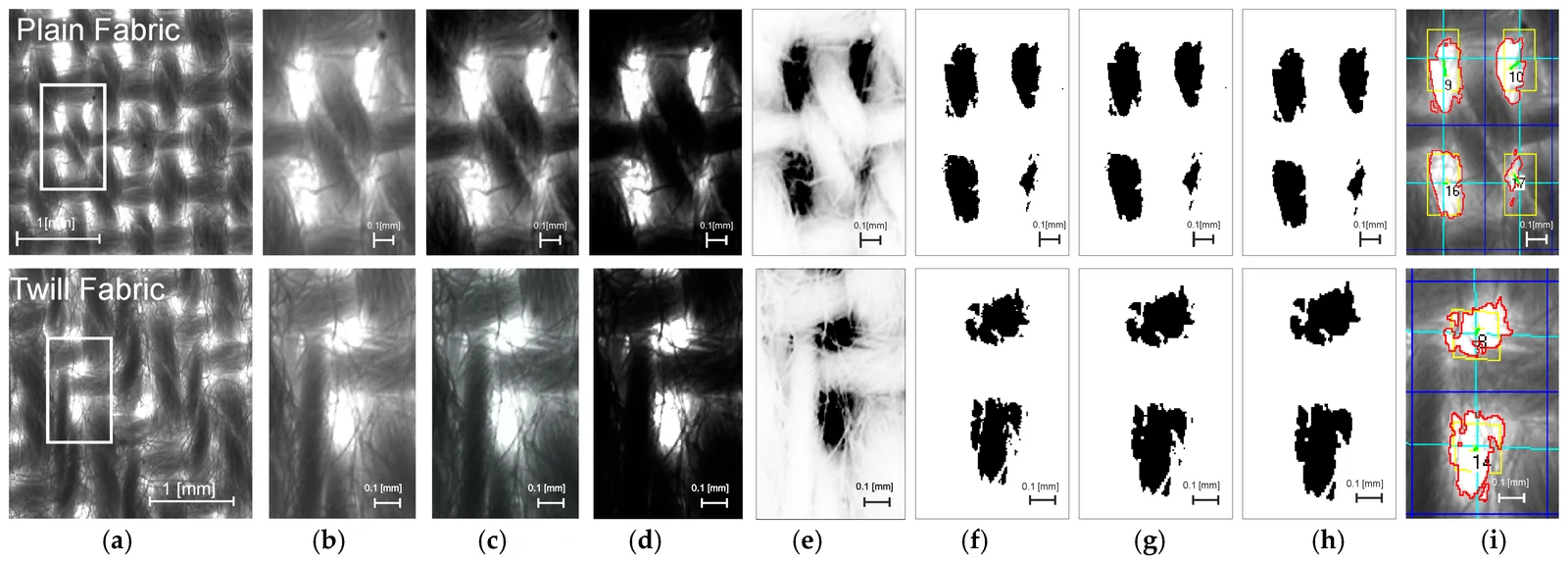
Algorithms for processing and morphometric image analysis: (**a**) raw image after acquisition; (**b**) low-pass filtering; (**c**) histogram equalization; (**d**) non-linear filtration (x^2^); (**e**) image negation; (**f**) thresholding; (**g**) morphological closing; (**h**) morphological opening; (**i**) cluster analysis for segmentation, recognition, classification, and interpretation of ITPs using a proprietary procedure.

Image preprocessing, segmentation, recognition, classification, and morphometric analysis were conducted using the proprietary MagFABRIC 2.1. software (developed by the author and used at Lodz University of Technology, Lodz, Poland). This software was specifically designed for the structural analysis of woven fabrics, particularly those made from spun yarns, which are characterized by hairiness, variable diameter, and blurred boundary line that can complicate inter-thread pore (ITP) segmentation. The software includes dedicated preprocessing steps to minimize the impact of such noise on pattern recognition. It enables semi-automatic detection of pattern repeats, quantification of intra- and inter-repeat inhomogeneity, and extraction of structural parameters based on texture segmentation and grayscale variation. The software integrates algorithmic modules for image enhancement, cluster recognition, morphometric analysis, and statistical evaluation—all tailored to research on fabric regularity and autoregulatory phenomena. The preprocessing included low-pass filtering, histogram equalization (0–255 gray levels), quadratic filtering, image negation, thresholding, and morphological operations (closing and opening). Proper image preprocessing is crucial for optimizing the ITP detection algorithms used in morphometric analysis (Figure 4) [27].

Thresholding played a key role in this segmentation process. Automatic thresholding, tailored to the specific characteristics of each image, enabled the effective separation of the foreground (ITPs) from the background. The threshold used was generated using two region-splitting methods that utilized the intensity distributions of the background p(x, y) and the object f(x, y) within each image. The segmentation algorithm, incorporating both Gaussian and Poisson models, supported the determination of the optimal threshold value. This dual-model approach also served as an internal cross-validation mechanism, enhancing the robustness and consistency of the segmentation across the dataset [27].

ITP identification and segmentation were performed using cluster analysis. A multistep approach was applied, considering the structural features of the fabric and their spatial distribution. Pixels belonging to the same clusters were grouped into ITPs, which were then organized into columns and rows. The segmented ITPs were analyzed to extract key morphometric parameters such as size, shape, and location [28].

Next, individual structural parameters were assigned to the weave repeat based on the principle of identifying the smallest structural elements and weave parameters. This method provides an optimized framework for precise ITP identification and special location relative to the thread pitch in fabric images, enabling high-throughput structural analysis, even in cases where ITPs are minimally visible [28].

### 2.5. Method for Assessing the Homogeneity of the Fabric Structure

The primary objective of image analysis is to evaluate the homogeneity of the fabric structure by introducing a novel approach that assesses this parameter in terms of intra-repeat (IAR) and inter-repeat (IER) homogeneity in woven fabric structures.

**Figure 5:**
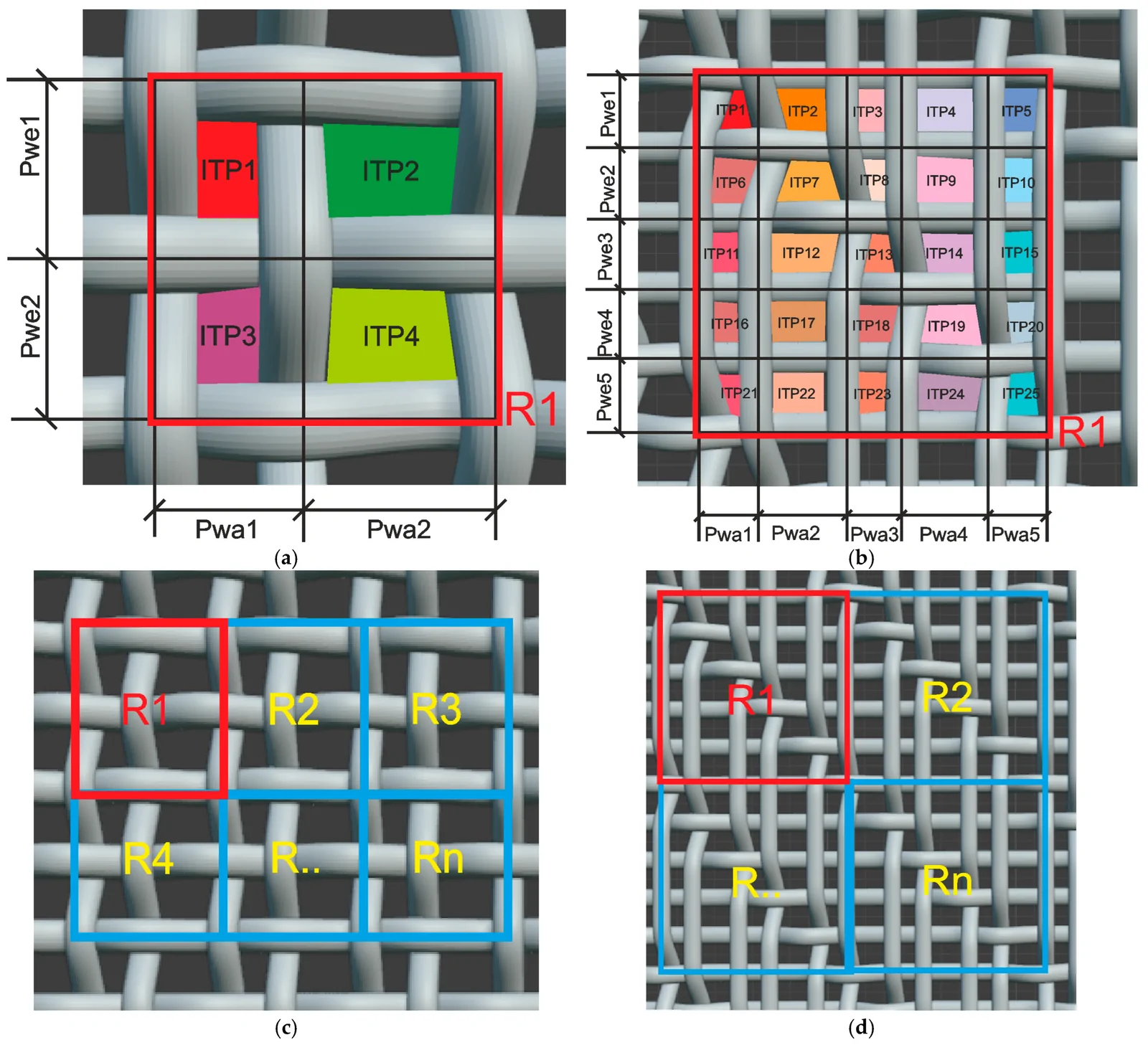
Visualization of the method for assessing the homogeneity of the fabric structure: (**a**) elementary unit—weave repeat R1 with ITPs in plain weave; (**b**) elementary unit—weave repeat R1 with ITPs in twill weave; (**c**) set of the units—weave repeats [R1–Rn] in plain weave; (**d**) set of the units—weave repeats [R1–Rn] in twill weave.

In this method, IAR homogeneity is defined as the repeatability of structural parameters—including the size, shape, and location of ITPs, as well as the values and positions of warp and weft thread pitches—within individual elementary units of the weave repeat [R_1_–R_n_], as illustrated in Figure 5.

In contrast, IER homogeneity refers to the consistency of the same structural parameters across the entire fabric structure, precisely assigned and sorted according to weave report components within a set of elementary units (weave repeats) [R_1_–R_n_].

While IAR homogeneity effectively reflects intra-repeat variability, IER homogeneity is for analyzing inter-repeat interference, particularly when caused by random external factors or structural irregularities between repeats, such as differences in fabric width or specific repeat properties. Together, IAR and IER homogeneity provide a comprehensive understanding of structural variability.

Structural parameters for all analyzed fabrics were assigned to individual weave repeats. Subsequently, coefficients of variation were determined for IAR and IER variability, enabling a quantitative assessment of fabric structure homogeneity. For each structural parameter assigned to specific weave repeat sections, the coefficient of variation was calculated using the general formula:

(2) $$V=\frac{\delta }{\bar{x}}\cdot 100\%$$

where *δ*—standard deviation, and $\bar{x}$ —mean value.

To evaluate the inhomogeneity of fabric structure within IAR and IER, the following variability indices were defined:

- Intra-repeat inhomogeneity ( ${\bar{V}}_{IAR}$ ),
- Inter-repeat inhomogeneity ( ${\bar{V}}_{IER}$ ),
- Global inhomogeneity ( $\bar{V})$.

These coefficients were calculated based on the variability of structural parameters, including the size, shape, and location of ITPs, as well as warp and weft pitches and their deviations from the average structural grid of the textile. The weighting coefficients (0.2 and 0.1) were derived from theoretical assumptions and validated through synthetic image modeling and multiple regression analysis, confirming their optimal impact on the model’s correlation with structural inhomogeneity. The formulas for these coefficients are presented below:

(3) $${\bar{V}}_{IAR}=0.2{\cdot V}_{{IAR}_{A}}+0.2{\cdot V}_{{IAR}_{S}}+0.2{\cdot V}_{{IAR}_{P_{wa}}}+0.2{\cdot V}_{{IAR}_{P_{we}}}+0.1{\cdot V}_{{IAR}_{D_{ITP}}}+0.1{\cdot V}_{{IAR}_{RID}}\;\left[\%\right]$$

(4) $${\bar{V}}_{IER}=0.2{\cdot V}_{{IER}_{A}}+0.2{\cdot V}_{{IER}_{S}}+0.2{\cdot V}_{{IER}_{P_{wa}}}+0.2{\cdot V}_{{IER}_{P_{we}}}+0.1{\cdot V}_{{IER}_{D_{ITP}}}+0.1{\cdot V}_{{IER}_{RID}}\;\;\;\;\;\;\left[\%\right]$$

(5) $$\bar{V}={\bar{V}}_{IAR}+{\bar{V}}_{IER}\;\left[\%\right]$$

where

- ${\bar{V}}_{IAR}$, ${\bar{V}}_{IER}$ —intra-repeat and inter-repeat inhomogeneity indices [%],
-
${\;V}_{{IAR}_{A}}$ , $V_{{IER}_{A}}-$ inhomogeneity of ITPs area,
-
$V_{{IAR}_{S}}$ , $V_{{IER}_{S}}$ —inhomogeneity of ITPs shape, calculated as
(6) S=0.45⋅Feret+0.45⋅AspecR+0.1⋅FormF[−]
  with
  − Feret = W/H—elongation, (width to height ratio),
  − AspectR = D_MIN_/D_MAX_—ovality (minor to major diameter ratio),
  − FormF = $\frac{4\Pi \cdot A}{L^{2}}$—form factor (edge complexity), where A—area, and L—perimeter,

- $V_{{IAR}_{P_{wa}}}$ $V_{{IER}_{P_{wa}}}{,V}_{{IAR}_{P_{we}}}$ , $V_{{IER}_{P_{we}}}$ —inhomogeneity of warp and weft thread pitches,
-
$V_{{IAR}_{D_{ITP}}}$ , $V_{{IER}_{D_{ITP}}}$ —inhomogeneity of the distance from the ITPs center to the nearest intersection of the average grid, calculated as
(7) $$D_{\mathrm{ITP}}=\frac{\bar{Pr}-C}{\bar{Pr}};\;\bar{Pr}=\sqrt{\bar{{P_{wa}}^{2}}+\bar{{P_{we}}^{2}}}$$
  where C—ITP center coordinate, and

$\bar{Pr}$ – diagonal of the P_wa_ and P_we_ pitches.

-
$V_{{IAR}_{RID}}$ , $V_{{IER}_{RID}}$ inhomogeneity of relative area, defined as
(8) $$\mathrm{RID}=\frac{A}{\bar{A_{IDE}}}$$
  where ${\bar{A}}_{IDE}\;$ —average ideal grid area [29].

**Figure 6:**
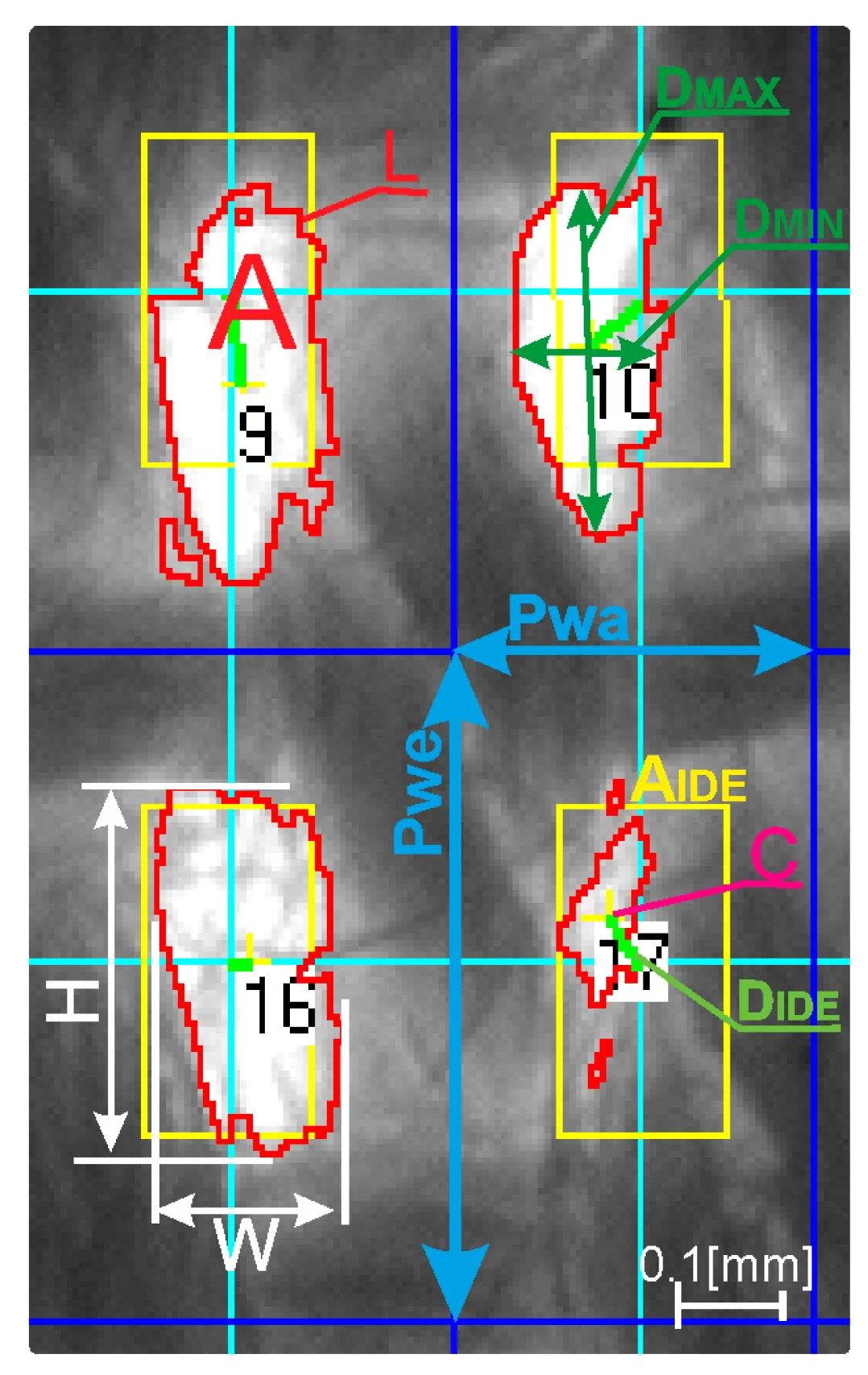
Morphometric parameters of ITPs: A—area, L—perimeter, D_MAX_, D_MIN_—maximum and minimum diameters, H and W—height and width, C—center of gravity, ${\bar{A}}_{IDE}$ —average ideal area, D_ITP_—distance from C to grid crossing, P_wa_, P_wa_ warp, and weft thread pitches.

Figure 6 shows the morphometric parameters applied in the quantitative assessment of inter-thread pores (ITPs), which form the basis for subsequent structural homogeneity analysis. 

### 2.6. Method for Air Permeability Testing

**Figure 7:**
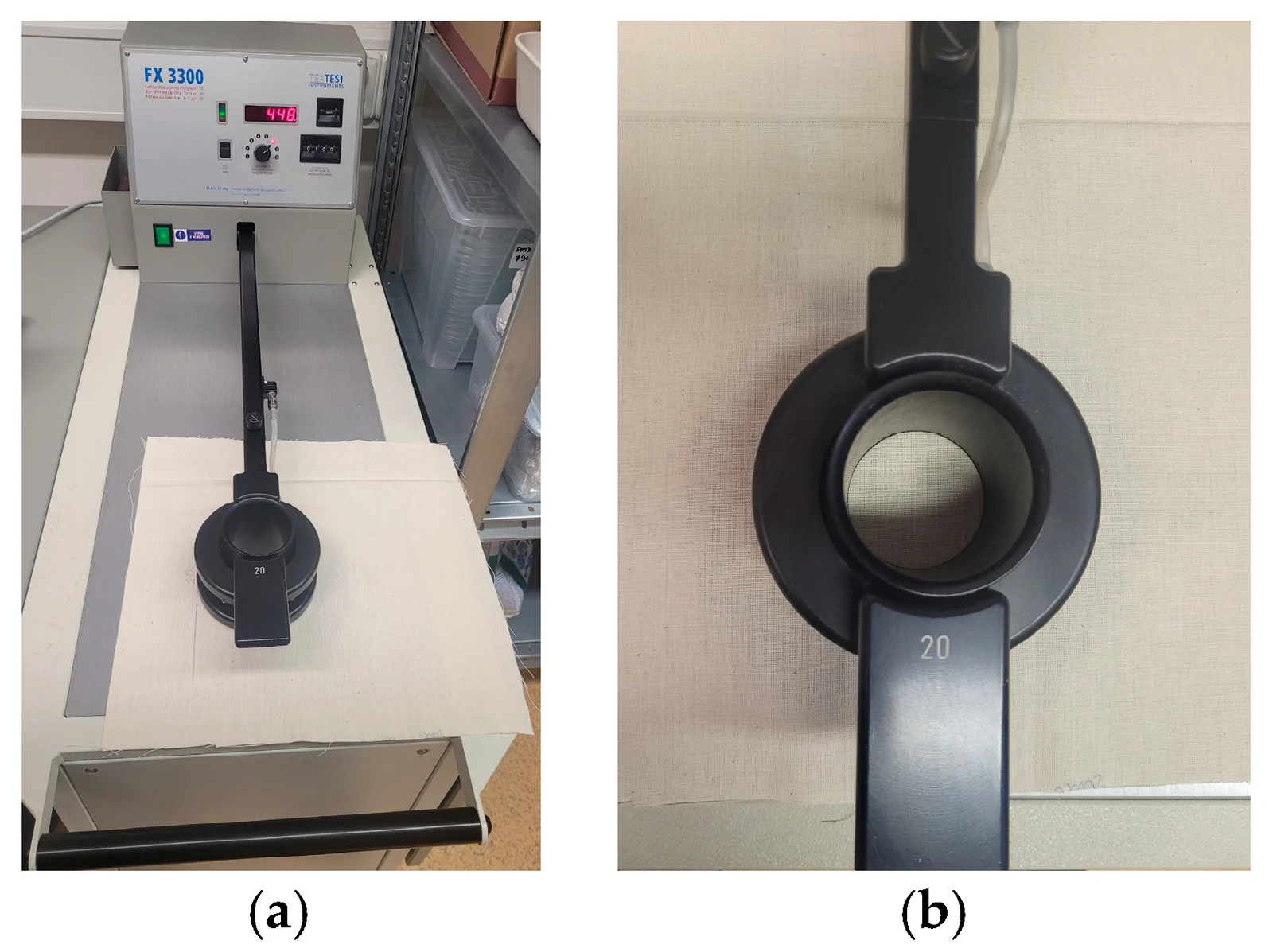
FX 3300 Air Permeability Tester III used for the measurement of airflow through woven fabrics: (**a**) general view of the testing device; (**b**) detailed view of the 20 mm^2^ circular testing head.

To evaluate the relationship between the structural homogeneity of fabrics and their functional performance, air permeability tests were conducted for all analyzed samples. The measurements were carried out using the FX 3300 Air Permeability Tester III (TEXTEST Instruments, Schwarzenbach, Switzerland) (Figure 7), in accordance with the CEN (2024) [32] and ASTM (2018) [33] standards. Each fabric was tested under controlled laboratory conditions, using a circular testing head with a measurement area of 20 mm^2^. The device applied a constant pressure differential across the fabric surface to determine the airflow rate passing through the material. The obtained air permeability values were expressed in mm/s and used for further correlation with structural homogeneity indices derived from image analysis.

### 2.7. Multiple Regression Analysis Method

To analyze the influence of weaving process parameters and environmental factors on the structural homogeneity of fabrics, a statistical approach based on multiple linear regression was applied. The analysis was performed using Statistica software (StatSoft Polska, available at: https://www.statsoft.pl/en/software/statistica/), employing a stepwise regression method to identify the most significant predictors influencing the homogeneity indices. The regression models were developed separately for plain and twill weave fabrics groups to reflect their structural characteristics. Both linear and nonlinear dependencies were considered by incorporating polynomial and interaction terms. The dependent variables in the models were structural homogeneity indices obtained from image analysis, while the independent variables included selected weaving process parameters and environmental conditions recorded during fabric production. Model quality and predictor relevance were evaluated using the F-test at a 95% confidence level. To ensure clarity and avoid overfitting models with higher-order terms, the number of input variables was reduced to the ten most statistically significant predictors (F = 10). 

## 3. Results

### 3.1. Results from the Experimental Plan

As part of the experimental plan for the group of plain weave fabrics, 30 out of 31 planned samples were successfully produced. Sample no. 17 could not be completed due to technical limitations associated with the extreme value of shed closure timing (Xp_1_ = 388.8°). However, all variants using a shed closure angle of 374.4° in the open shed configuration were realized, thus enabling the fabrication of samples with all three shed configurations: open, closed, and crossed.

For the group of twill weave fabrics woven on the SACM-MAV Rp-C-180 loom, the available adjustment range for weaving parameters was significantly narrower than that of the Saurer W100 loom used for the plain weave fabrics. The adjustable shed closure timing (Xt_2_) on this loom allowed for only closed and crossed shed configurations. Despite this limited range, all planned combinations of process parameters were implemented.

The resulting values of structural uniformity $({\bar{V}}_{IAR}intra-repeat\;inhomogeneity$ , ${\bar{V}}_{IER}$ inter-repeat inhomogeneity, and global inhomogeneity $\bar{V}$ ) as well as air permeability (AirF), for both plain weave (uP) and twill weave (uT) fabrics, which are summarized in Table 3.

### 3.2. Structural Changes in Woven Fabrics

During the experimental process, structural differences were observed in both plain and twill weave fabric groups. In the majority of samples, the reed mark was distinctly visible due to threading two warp yarns through each split of the reed and heald eye. However, in several cases, the reed mark diminished or disappeared entirely, resulting in a more uniform structure. This phenomenon was particularly evident in the plain weave group.

In the twill weave fabrics, structural changes were also observed, depending on the yarns used with different twist directions, especially on the warp. Fabrics woven with S-twist yarns—characterized by lower mass per unit length but greater diameter and hairiness—exhibited a denser structure with a more pronounced twill pattern. In some cases, partial disappearance of the reed mark and increased structural uniformity were also achieved, particularly in samples using alternating S- and Z-twist warp yarns.

**Figure 8:**
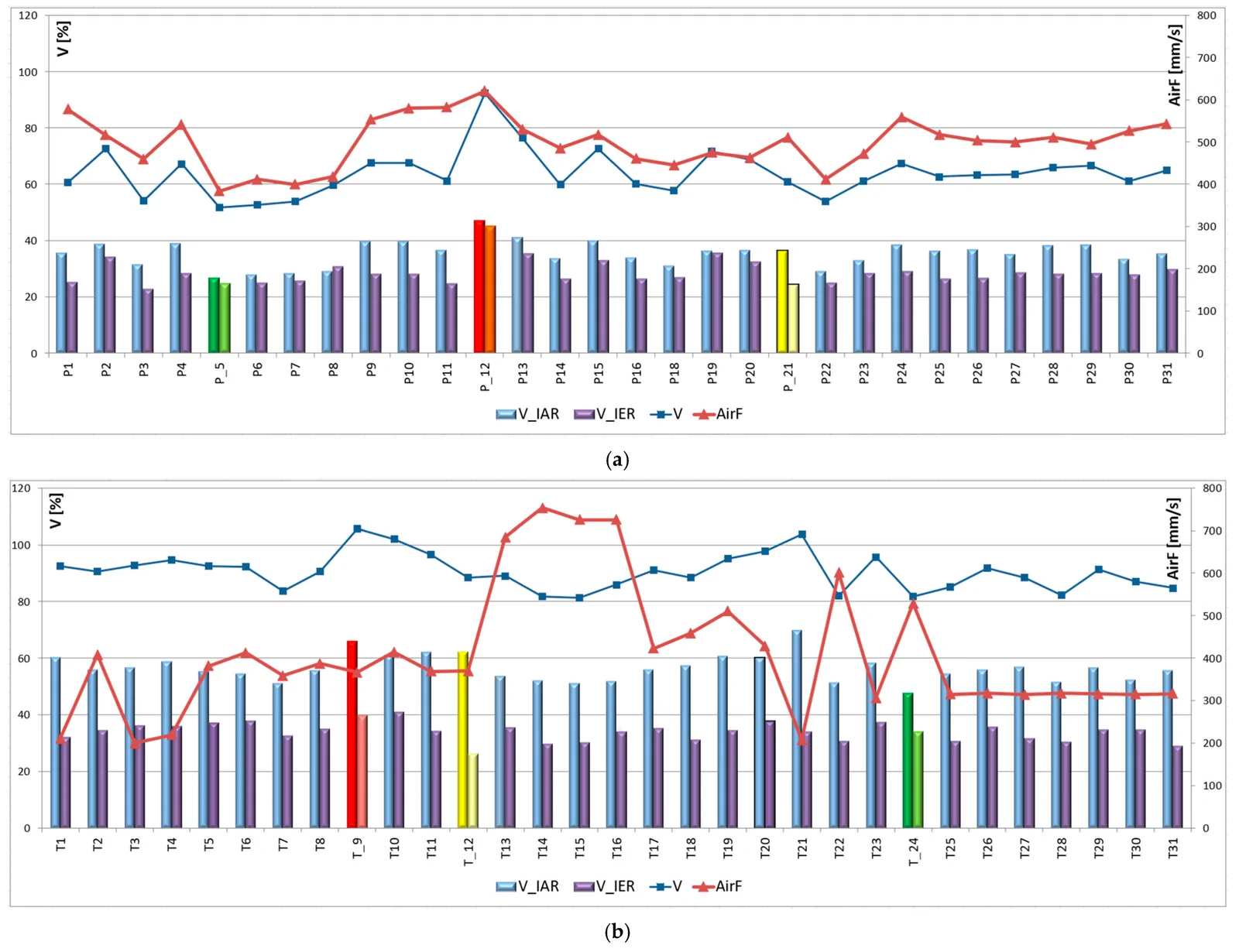
Visualization of the results of the fabric structure inhomogeneity and the air flow analysis, where ${\bar{V}}_{IAR}$ —intra-repeat inhomogeneity, ${\bar{V}}_{IER}$ —inter-repeat inhomogeneity, $\bar{V}$ —global inhomogeneity, AirF—air flow: (**a**) plain weave fabrics, (**b**) twill weave fabrics.

**Figure 9:**
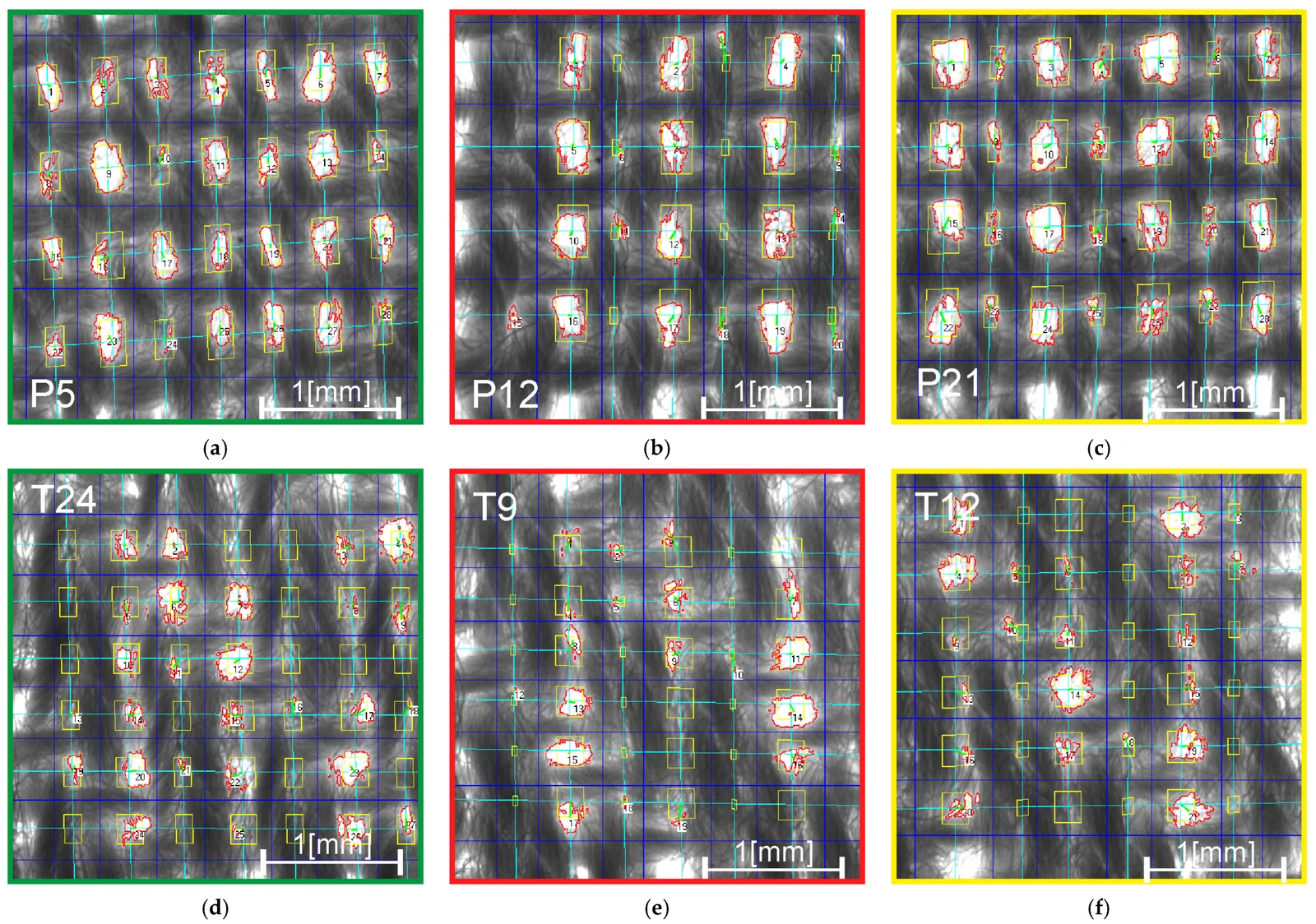
Visualization of three characteristic fabric structures with different levels of inhomogeneity ${\bar{V}}_{IAR}$ S, ${\bar{V}}_{IER,}$ $\bar{V}$ after image analysis. Plain weave fabrics: (**a**) P5, (**b**) P12, (**c**) P21. Twill weave fabrics: (**d**) T9, (**e**) T12, (**f**) T24.

The results of the uniformity parameter analysis $({\overset{-}{V}}_{IAR}$ , ${\bar{V}}_{IER}$ , $\bar{V}$ ) revealed a wide range of inhomogeneity levels. The values ranged from 24.91 (P5) and 26.20 (T12) to 47.13 (P12) and 65.96 (T9), respectively. Samples were classified according to a color-coded system: green indicating low values, yellow intermediate values, and red high values, as shown in Figure 8 and Figure 9, the latter of which shows representative images of three characteristic fabric structures.

Under favorable weaving conditions, it was possible to produce fabrics with no visible warp grouping (e.g., P5) or partial disappearance of the reed mark (e.g., T24), corresponding to low ${\bar{V}}_{IAR}$ , and ${\bar{V}}_{IER}$ values (green group). Samples P21 and T12 (yellow group) exhibited low inter-repeat inhomogeneity ( ${\bar{V}}_{IER}$ ) but higher intra-repeat variation ( ${\bar{V}}_{IAR}$ R). The red group, including samples P12 and T9, showed the highest levels of both ${\bar{V}}_{IAR}$ and ${\bar{V}}_{IER}$ , indicating significant structural irregularities across and within repeat units.

### 3.3. Results of the Regression Analysis

To determine the influence of weaving parameters (Xp_1_–Xp_4_, Xt_1_–Xt_4_) and environmental conditions (T—temperature, H—humidity, P—atmospheric pressure) on the structural inhomogeneity of woven fabrics, multiple regression models were developed separately for plain weave (uP) and twill weave (uT) fabric groups. Both linear and nonlinear relationships were considered through the inclusion of polynomial and interaction terms (up to the third degree), resulting in a total of 43 input variables. The dependent variables included ${\bar{V}}_{IAR}$ , ${\bar{V}}_{IER}$ , and $\bar{V}$ —global inhomogeneity. The regression analysis was performed using the stepwise progressive method in the Statistica software. To explore the structure of dependencies at higher levels of model complexity, the number of predictors was limited to the 10 most significant ones (F = 10). The results for plain weave fabrics are summarized in Table 4.

For the models developed at F = 10, all four mechanical loom settings—shed closure timing (Xp_1_), lease rod position (Xp_2_), backrest roller position (Xp_3_), and warp pre-tension (Xp_4_)—were found to significantly influence fabric uniformity.

For intra-repeat inhomogeneity ( ${\bar{V}}_{IAR}$ ), a significant effect was observed for the interaction (Xp_1_^2^∙Xp_4_) (Std. BETA = 0.22, t = 6.15, *p* < 0.0000), which was further influenced by the third-order humidity term H^3^, whereas the cubic component of Xp_3_^3^ and the linear term of humidity H acted as stabilizing factors.

The model for inter-repeat inhomogeneity ( ${\bar{V}}_{IER}$ ) demonstrated a strong fit (R^2^ = 0.74). The most influential predictors included complex interactions of mechanical parameters, such as

(Xp_1_∙Xp_2_∙Xp_4_) (Std. BETA = 17.04, t = 16.61, *p* < 0.0000),
(Xp_2_∙Xp_4_) (Std. BETA = –16.98, t = −16.94, *p* < 0.0000),
(Xp_3_^2^∗Xp_2_) (Std. BETA = 7.28, t = 13.69, *p* < 0.0000),

As well as single-variable effects:

Xp_2_ (Std. BETA = −9.54, t = −19.07, *p* < 0.0000),
Xp_2_^3^ (Std. BETA = 4.54, t = 18.48, *p* < 0.0000),
Xp_3_^3^ (Std. BETA = −3.88, t = −12.97, *p* < 0.0000).

Nonlinear effects of temperature and humidity (T^3^, T^2^, H, H^2^) also played an important role. The opposing signs of the coefficients suggest the existence of an optimal environmental range for maintaining fabric homogeneity.

The model for global inhomogeneity ( $\bar{V}$ ) revealed similar relationships: the interaction (Xp_1_^2^∙Xp_4_) (Std. BETA = 0.12, t = 3.58, *p* < 0.0003) and the cubic term of humidity H^3^ (Std. BETA = 1.29, t = 7.65, *p* < 0.0000) were associated with increased variability, while Xp_3_^3^ (Std. BETA = −0.37, t = −11.50, *p* < 0.0000) and the linear term of humidity H contributed to structural stabilization.

These results highlight the nonlinear and complex impact of both environmental conditions and process parameters on fabric structure. The findings confirm that, for plain weave fabrics, structural uniformity is not governed by any single loom setting but rather by the precise configuration and mutual interactions of the key loom parameters: shed closure timing, lease rod position, backrest roller position, and warp pre-tension. Furthermore, environmental conditions such as humidity and temperature exert nonlinear moderating effects, which may either intensify or mitigate influences depending on their specific combination.

For twill weave fabrics (uT), the regression models developed at F = 10 showed that the direction of twist in the warp yarn Xt_3_ had the most significant influence on structural inhomogeneity (see Figure 5).

Table 5 shows the final regression models for twill weave fabrics.

For intra-repeat inhomogeneity ( ${\bar{V}}_{IAR}$ ), the model showed a moderate fit (R^2^ = 0.35) and identified two statistically significant predictors: the warp yarn twist direction Xt_3_, which had a positive influence (Std. BETA = 0.57, t = 19.75, *p* < 0.0001), and the quadratic component of humidity H^2^, which acted as a stabilizing factor (Std. BETA = −0.14, t = −4.78, *p* < 0.0001). This suggests that an increased twist in the warp yarn leads to greater variability within structural repeats, while moderate humidity levels may help mitigate this effect.

In the case of inter-repeat inhomogeneity ( ${\bar{V}}_{IER}$ ), only the variable Xt_3_ was statistically significant, with a negative impact (Std. BETA = −0.17, t = −4.74, *p* < 0.0001), although the overall model fit was relatively low (R^2^ = 0.03). This indicates that greater twists in warp yarn reduce variability between structural repeats.

The model for global inhomogeneity ( $\bar{V}$ ) mirrored the findings for intra-repeat variability, with a similarly moderate fit (R^2^ = 0.34) and a strong positive effect of warp yarn twist direction Xt_3_ (Std. BETA = 0.58, t = 6.95, *p* < 0.0001), confirming the role of this variable as the dominant mechanical factor influencing the uniformity of twill fabrics.

Overall, the regression analysis for twill weaves indicates that the direction of twist in the warp yarn Xt_3_ is the key determinant of structural homogeneity. Its effect varies depending on the type of inhomogeneity considered—amplifying intra-repeat and global irregularities while reducing inter-repeat differences. Additionally, environmental humidity, particularly its nonlinear components, interacts with mechanical settings, further influencing the woven fabric structure.

## 4. Discussion

### 4.1. Interpretation of Results

The analysis of two fabric groups—plain weave and twill weave—reveals clear differences in the mechanisms governing structural homogeneity and the fabrics’ capacity for autoregulation. Three distinct structural categories were identified: green (P5 and T24), red (P12 and T9), and yellow (P21 and T12). For each group, specific configurations of weaving process parameters enabled structural autoregulation to varying degrees. These parameter sets are summarized in Table 6.

The multiple regression models demonstrated that fabric structural uniformity is influenced by the interaction of multiple parameters—mechanical and environmental. This indicates the potential for achieving a state of structural autoregulation, defined as the configuration in which disturbances such as warp yarn clustering are compensated by stabilizing mechanisms operating within the fabric structure. Achieving this balance is possible when a specific combination of process parameters is present—particularly Xp_1_, Xp_4_, Xt_3_, and Xt_2_, which interact to create conditions favorable to uniform yarn alignment within the structure.

In the second experiment, involving twill weave fabrics, additional process variables were introduced: warp and weft yarn twist direction (Xt_3_ and Xt_4_). The results revealed that yarn twist is a dominant factor with a strong influence on the structural formation and the fabrics’ susceptibility to autoregulation. The F = 10 regression models with the strongest correlations showed that variation in Xt_3_ and Xt_4_ significantly overshadowed the effects of other process variables (such as Xp_1_ and Xp_2_), thereby limiting the capacity to compensate for local structural irregularities. Twist introduces internal stresses and deformations within the yarns, which largely determine yarn positioning in the fabric structure and consequently impose the character of that structure, reducing its self-regulatory potential.

Additionally, the analysis of the air permeability parameter (AirF) revealed that it is also strongly affected by yarn construction, particularly twist. In plain weave fabrics with constant yarn twists, AirF values were relatively consistent with the observed structural uniformity and may serve as a supportive indicator for evaluating structural regularity. However, in twill weave fabrics with variable yarn twists, these relationships were disrupted. In such cases, AirF no longer reliably reflects true structural uniformity, as its values are more strongly influenced by yarn geometry.

Therefore, under conditions involving variability in yarn construction parameters—especially twist—the proprietary inhomogeneity indicators ${\bar{V}}_{IAR}$ , ${\bar{V}}_{IER}$ , and $\bar{V}$ are far more appropriate tools for assessing fabric structural quality. These indicators enable objective and precise evaluation of structural uniformity regardless of raw material variability and reveal mechanisms that conventional parameters such as AirF are unable to capture.

However, the main sources of error in image-based assessment of structural uniformity may include segmentation inaccuracies caused by yarn hairiness and shading, optical distortions due to the lens or light shadows, and local variations in weave density across the fabric width. Detection of ITPs can also be hindered by yarn clustering in, for example, twill or satin weaves, which reduces their visibility. The observed higher ${\bar{V}}_{IAR}$ values for twill weaves (47.77–65.96%) compared to plain weaves (26.74–47.13%) reflect intrinsic structural characteristics of the weave, such as longer, loosely interlaced yarns, which often reduce the visibility of ITPs. Based on repeatability tests and visual inspection of the segmentation results, the uncertainty associated with imaging and processing artifacts, as well as the ITPs visibility, is estimated not to exceed 3–5% depending on weave type and does not affect the relative conclusions drawn for different fabric types.

Referring to the recent scientific literature, structural analysis related to porosity in woven fabrics has used image thresholding and segmentation [7,8], while machine learning has supported loop geometry analysis in knits to predict cover factor [30]. Classical texture-based methods—such as gray-level co-occurrence matrices (GLCM), Fourier transforms, and Gabor filters—are still used for defect detection and structural evaluation [34,35]. More advanced approaches include contourlet-domain feature learning [36], 3D porosity mapping [37], and deep learning models such as CNNs and Mask-RCNNs, further enhanced by multi-cue aggregation strategies [35,38,39]. Although effective for identifying surface-level features, these methods do not quantify microscale structural inhomogeneities. In contrast, the method proposed in this study enables high-resolution morphometric analysis of individual inter-thread pores, supporting both local and global assessment of pore location, distribution, structural arrangement in the weave repeat area, and subtle irregularities. This allows for identifying autoregulatory effects linked to weaving parameters and environmental conditions, offering a more process-relevant and physically grounded evaluation of fabric quality and structural inhomogeneity mechanisms.

### 4.2. Autoregulation Mechanisms

Structural autoregulation in woven fabrics, as proposed by Nosek [25], manifests through the gradual compensation of disturbances over successive weft insertions. The experimental results confirm the presence of this mechanism, notably through two feedback types: external (e.g., reed, backrest, warp tension) and internal (structural parameters e.g., yarn twist effects).

Nosek formulated an equilibrium equation that must always be satisfied at the fabric edge to maintain a stable structural state. This expression describes the interdependence of three parameters: X—beat-up zone length, Q_o_—initial warp tension, and $\phi_{o}$ —beat-up angle. Although the original equation is complex, it was linearized for practical application, resulting in a simplified linear relationship expressing the equilibrium between the relative changes in these three fundamental parameters of fabric structure:

(15) $$\phi_{o}-\gamma \cdot \;q_{o}+\zeta \cdot \;\chi =0$$

When a structural disturbance occurs due to a change in X, Q_o_, or $\phi_{o}$ , the woven structure attempts to return to a stable state. However, this autoregulation requires time, i.e., a certain number of wefts in section cycles.

The weaving process parameters identified through regression models were interpreted using Nosek’s autoregulation mechanism. The analysis was first performed for plain weave fabrics, where external feedback mechanisms played a particularly important role.

H↑↓:

The strongest influence on ${\bar{V}}_{IAR}$ is exerted by air humidity H. The negative correlation between H and intra-repeat inhomogeneity results from its impact on the coefficient of friction μ: as H increases, μ decreases. According to Nosek’s equation for minimum warp tension Q_omin_, a decrease in μ leads to a reduction in Q_omin_. Therefore, humidity significantly affects the autoregulation process within ${\bar{V}}_{IAR}$ , ${\bar{V}}_{IER}$ , and $\bar{V}.$ As H increases, structural homogeneity improves.

(Xp_1_^2^∙Xp_4_) ↓:

The interaction between Xp_1_ (shed closure timing) and Xp_4_ (warp pre-tension) is reflected in Nosek’s theory. Xp_1_ is directly related to the beat-up angle *ϕ*_b_ and warp tension. Initially, as *ϕ*_b_ increases (up to approx. 30°), warp tension Q_o_min also increases due to enhanced yarn interlocking. Beyond that point, greater yarn intersection enables more efficient beat-up, reducing Q_o_min and improving yarn arrangement.

Xp_2_ ↑↓:

In the regression model for ${\bar{V}}_{IER}$ , variable Xp_2_ (lease rod position) appears in interactions with several other parameters. Nosek noted that Xp_2_ influences warp length L_o_ within the shed, which in turn affects Q_o_. The elasticity constants for warp C_o_ and fabric C_f_ are inversely proportional to their respective lengths. To reduce Q_o_, one may either increase the working fabric length L_f_ or decrease the working warp length L_o_, which can be achieved by adjusting the lease rod position.

(16) $$\frac{C_{o}}{C_{f}}=\frac{E_{o}F_{o}}{E_{f}F_{f}}\cdot \frac{L_{f}}{L_{o}}=K\cdot \frac{L_{f}}{L_{o}}$$

Xp_3_ ↓:

In all models, Xp_3_ (backrest roller position) exhibits a negative correlation with the inhomogeneity indices. A lower roller position increases tension in the upper warp branch (with the hold of the weft) and decreases it in the lower branch. This tension differential reduces friction and facilitates structural autoregulation.

For twill weave fabrics, the regression analysis indicated that internal feedback mechanisms predominated, with twist direction Xt_3_ and humidity H emerging as the key variables.

Xt_3_↑↓:

Twist direction of the warp yarn (Xt_3_ with configurations such as S/S, S/Z, and Z/Z) has the most significant effect on ${\bar{V}}_{IAR},$ ${\bar{V}}_{IER}$ , and $\bar{V}$ . The interaction between matching or opposing twist directions influences the friction coefficient μ, thereby affecting the efficiency of autoregulation. Yarn twist governs the behavior of adjacent threads in contact—either stabilizing or disrupting the local structure.

H↓:

Humidity also affects ${\bar{V}}_{IAR}$ in twill fabrics, similarly to plain weaves, through its effect on the friction coefficient μ.

## 5. Conclusions

- A significant influence of weaving process parameters and atmospheric conditions on the structural homogeneity of fabrics was demonstrated at the intra-repeat ${\bar{V}}_{IAR}$ , inter-repeat ${\bar{V}}_{IER}$ , and global $\bar{V}\;$ levels in the regression models developed at F = 10. For plain weave fabrics, all four mechanical loom settings—shed closure timing Xp_1_, lease rod position Xp_2_, backrest roller position Xp_3_, and warp pre-tension Xp_4_—were correlated with structural homogeneity. For twill weave fabrics, regression models indicated that the warp yarn twist direction Xt_3_ played a dominant role in autoregulation near Xt_1_ backrest roller position, Xt_2_ shed closure timing, and Xt_4_ weft yarn twist directions. Atmospheric conditions also influenced the outcomes, particularly relative humidity H, which demonstrated significant interactions in both fabric groups.
- Specific configurations of weaving parameters were identified that result in the highest and lowest structural homogeneity in plain- and twill-woven cotton fabrics, providing a foundation for the purposeful design of fabric properties. For plain weave, the highest homogeneity for P5 fabric was achieved with Xp_1_ = 374.4°, Xp_2_ = 102.5 cm, Xp_3_ = 88 cm, and Xp_4_ = 12.76 cN/thread. In twill weave, the highest homogeneity occurred under conditions for T24 fabric of Xt_1_ = 104 cm, Xt_2_ = 360°, Xt_3_ = S; Z, and Xt_4_ = Z.
- The phenomenon of structural autoregulation was observed under real weaving conditions, confirming the system’s ability to compensate for disturbances. The weaving parameters showing the strongest correlations included, for plain weave: humidity H and the interaction (Xp_1_·Xp_4_), Xp_2_, Xp_3_; and for twill weave: Xt_3_ and H.
- The results confirm that structural autoregulation can substantially affect fabric homogeneity and, consequently, its functional properties—such as filtration efficiency. For example, plain weave fabrics with the lowest inhomogeneity ( $\bar{V}$ = 51.66%) exhibited the lowest air flow (AirF = 383.73 mm/s). For twill fabrics, the AirF level does not reflect the level of structural uniformity because yarn geometry is a confounding factor. Therefore, AirF cannot be used to assess uniformity for every type of weave fabric.
- Optimization of the weaving process is a key factor in achieving structural homogeneity and, therefore, reproducible properties in technical textiles, including filtration fabrics, composites, and materials with controlled heterogeneity. Adjusting key parameters—especially warp tension and shed closure timing—allowed global inhomogeneity to be reduced by 41 or 21 across fabric variant groups, e.g., $\bar{V}$ (51.66–92.33%) for plain and $\bar{V}$ (81.74–105.66%) for twill weave.
- The study demonstrates that specific weaving parameters—such as backrest roller position, shed closure timing, warp tension, and yarn twist direction—have a substantial impact on fabric structural homogeneity at intra-repeat, inter-repeat, and global levels. Regression models indicated that as much as 74% of the variance could be explained by these parameters in plain weave, and as much as 35% in twill weave.
- The homogeneity analysis enhances the current understanding of process–structure–property interactions. Autoregulation observed in plain and twill weaves indicates that woven structures are capable of self-regulating disturbances under favorable weaving conditions—an effect that requires further investigation, particularly for other weaves. Further research direction will examine how fabric structural homogeneity influences mechanical properties and the uniformity of stress distribution in textiles. These findings support the predictive design of specialized textiles (e.g., filters, composites) by linking process conditions to fabric properties. Future research should extend this approach to fabrics made from various raw materials, particularly synthetics, which may respond differently to climatic and weaving conditions than cotton due to differing friction coefficients.

## 6. Patents

The method of analyzing fabric homogeneity using image analysis has been patented by the Patent Office of the RP.

## Figures and Tables

**Table 1:** Yarn parameters were measured using USTER^®^ TESTER and compared with USTER^®^ STATISTICS 2018 * (50% reference value).

Yarns Parameter	Plain Fabrics		Twill Fabrics		USTER^®^ 50% Reference *
	Warp (A)	Weft (B)	Warp (C)	Weft (D)	
Linear mass, Tex	45.0	36.0	40.4	44.5	-
Twist per 1 m	646 S	604 S	585 S	582 Z	600–650
English cotton count, Ne	13.12	16.40	14.62	13.27	13–16
CVm (%) of linear mass	11.40	13.93	12.03	10.60	12.5–14.0
Hairiness (fibers per 1 m)	6.22	7.31	8.06	6.98	6.5–7.0
Thin places (−40%)/1000 m	0	60	4	0	0-10
Thick (+35%)/1000 m	80	572	293	99	100–600
Neps (+140%)/1000 m	280	445	419	178	100–400
Breaking force, cN	705	518	657	512	-
Tenacity, cN/Tex	15.66	14.38	16.26	11.51	13–16
Breaking elongation, %	4.41	5.38	12.88	6.5	4–10

* Source: USTER^®^ STATISTICS 2018. October 2018. Available online: https://www.uster.com/fileadmin/user_upload/Downloads/2_ValueAddesServices/Uster_Statistics/en_USTER_NewsBulletin51_final_WEB_LowRes.pdf (accessed on 24 March 2025). Note: S and Z refer to the twist direction of the yarn. CVm—coefficient of variation of mass; Ne—English cotton count; Tex—linear density in grams per 1000 m.

**Table 3:** Summary of the weaving process parameters applied in the experimental plan PS/DS-P:λ(λ) ${\hat{x}}_{k}$ (−α, −1, 0, 1, + α), where: α = 2. The core of the plan is N_k_ = 2^i^ = 2^4^ = 16, star points n_α_ = 2i = 2 × 4 = 8; and the number of central points is N_0_ = 7 (with N_0_ > 1). The axial distance for four factors is α_4_ = 0.86. The table also presents the results of the inhomogeneity and air permeability analysis, where: ${\bar{V}}_{IAR}:$ intra-repeat inhomogeneity, ${\bar{V}}_{IER}\;:$ inter-repeat inhomogeneity, $\bar{V}$ : global inhomogeneity, AirF [mm/s]: air flow rate. The results are provided separately for plain weave (uP) and twill weave (uT) fabrics. See the following section for a description of the input and output variables used in the regression models.

Plain		Input				Output				Twill	Input				Output			
	uP	Xp_1_	Xp_2_	Xp_3_	Xp_4_	${\bar{V}}_{IAR}$	${\bar{V}}_{IER}$	$\bar{V}$	AirF	uT	Xt_1_	Xt_2_	Xt_3_	Xt_4_	${\bar{V}}_{IER}$	${\bar{V}}_{IAR}$	$\bar{V}$	AirF
	-	[°]	[cm]	[cm]	[cN/ Thread]	[%]	[%]	[%]	[mm/s]	-	[cm]	[°]	-	-	[%]	[%]	[%]	[mm/s]
The core of the plan	P1	374.4	102.5	84	12.76	35.49	25.18	60.67	577.36	T1	102	330	S	S	32.30	60.24	92.54	210.09
	P2	345.6	102.5	84	12.76	38.57	34.11	72.68	516.45	T2	106	330	S	S	34.66	55.87	90.53	408.00
	P3	374.4	117.5	84	12.76	31.39	22.77	54.16	460.09	T3	102	310	S	S	36.14	56.53	92.67	199.45
	P4	345.6	117.5	84	12.76	38.86	28.34	67.21	541.18	T4	106	310	S	S	36.00	58.71	94.71	219.18
	P5	374.4	102.5	88	12.76	26.74	24.91	51.66	383.73	T5	102	330	Z	S	37.09	55.37	92.46	381.55
	P6	345.6	102.5	88	12.76	27.78	24.99	52.77	412.09	T6	106	330	Z	S	38.02	54.29	92.30	412.36
	P7	374.4	117.5	88	12.76	28.33	25.60	53.92	399.73	T7	102	310	Z	S	32.72	51.06	83.77	358.82
	P8	345.6	117.5	88	12.76	29.01	30.66	59.67	418.55	T8	106	310	Z	S	34.98	55.69	90.66	387.09
	P9	374.4	102.5	84	25.53	39.52	28.01	67.52	553.55	T9	102	330	S	Z	39.69	65.96	105.66	367.00
	P10	345.6	102.5	84	25.53	39.65	27.94	67.60	579.64	T10	106	330	S	Z	41.13	60.93	102.06	414.55
	P11	374.4	117.5	84	25.53	36.46	24.78	61.24	582.36	T11	102	310	S	Z	34.44	62.12	96.56	368.91
	P12	345.6	117.5	84	25.53	47.13	45.20	92.33	620.91	T12	106	310	S	Z	26.20	62.26	88.46	369.73
	P13	374.4	102.5	88	25.53	41.13	35.31	76.44	530.55	T13	102	330	Z	Z	35.45	53.61	89.06	685.18
	P14	345.6	102.5	88	25.53	33.48	26.44	59.92	485.82	T14	106	330	Z	Z	29.73	52.01	81.75	753.55
	P15	374.4	117.5	88	25.53	39.81	32.81	72.63	517.64	T15	102	310	Z	Z	30.24	51.11	81.35	725.55
	P16	345.6	117.5	88	25.53	33.73	26.32	60.05	460.55	T16	106	310	Z	Z	34.12	51.83	85.95	725.55
Star point	P17	388.8	110	86	19.15	-	-	-	-	T17	100	320	S;Z	S;Z	35.22	55.96	91.18	423.00
	P18	331.2	110	86	19.15	30.84	26.85	71.66	444.91	T18	108	320	S;Z	S;Z	31.20	57.21	88.41	459.27
	P19	360	90	86	19.15	36.27	35.39	68.74	475.09	T19	104	360	S;Z	S;Z	34.51	60.73	95.24	510.73
	P20	360	125	86	19.15	36.45	32.29	60.92	463.00	T20	104	303	S;Z	S;Z	37.59	60.12	97.71	428.09
	P21	360	110	82	19.15	36.40	24.52	53.88	510.73	T21	104	360	S	S;Z	34.09	69.69	103.78	206.91
	P22	360	110	90	19.15	28.96	24.92	61.02	411.64	T22	104	360	Z	S;Z	30.69	51.36	82.05	601.36
	P23	360	110	86	5.93	32.84	28.18	67.40	471.64	T23	104	360	S;Z	S	37.42	58.34	95.76	306.36
	P24	360	110	86	31.91	38.34	29.06	62.64	558.91	T24	104	360	S;Z	Z	33.97	47.77	81.74	528.55
Center of the plan	P25	360	110	86	19.15	36.24	26.40	63.28	517.64	T25	104	320	S;Z	S;Z	30.80	54.35	85.15	314.50
	P26	360	110	86	19.15	36.74	26.54	63.59	503.73	T26	104	320	S;Z	S;Z	35.81	55.92	91.73	317.25
	P27	360	110	86	19.15	35.08	28.51	65.98	500.18	T27	104	320	S;Z	S;Z	31.73	56.69	88.42	314.00
	P28	360	110	86	19.15	38.06	27.92	66.54	511.64	T28	104	320	S;Z	S;Z	30.62	51.55	82.17	316.75
	P29	360	110	86	19.15	38.25	28.29	61.12	495.27	T29	104	320	S;Z	S;Z	34.89	56.49	91.38	315.00
	P30	360	110	86	19.15	33.32	27.80	64.95	526.18	T30	104	320	S;Z	S;Z	34.81	52.20	87.00	314.50
	P31	360	110	86	19.15	35.36	29.60	60.67	543.55	T31	104	320	S;Z	S;Z	29.17	55.60	84.77	316.25

**Table 4:** Regression models for plain weave fabrics (uP). Dependent variables: ${\bar{V}}_{IAR}$ —intra-repeat inhomogeneity, ${\bar{V}}_{IER}$ —inter-repeat inhomogeneity, $\bar{V}$ —global inhomogeneity. Independent variables: Xp_1_—shed closure timing, Xp_2_—lease rod position, Xp_3_—backrest roller position, Xp_4_—pre-tension of the warp (F = 10).

F	Dependent Variable	R^2^	*R^2*	Most Significant Date(s) (Std. BETA, t, p)	Direction of Influence (Regression coeff. B)	
Model 1. ${\bar{V}}_{IAR}=$ 117.16 + (Xp_1_^2^∙Xp_4_) − (0.001∙Xp_3_^3^*) −* (1.026∙H) + (0.001∙H^3^)						(9)
10	${\bar{V}}_{IAR}$	0.27	0.26	Xp_1_^2^∙Xp_4_ (0.22, 6.15, 0.0000) Xp_3_^3^ (−0.42, −13.19, 0.0000) H (−1.03, −6.07, 0.0000) H^3^ (0.76, 4.48, 0.0000)	↑ (Xp_1_^2^·Xp_4_), H^3^ ↓ Xp_3_^3^, H	
Model 2. ${\bar{V}}_{IER}=\;$ 216.94 + (0.30∙H) − (0.004∙H^2^*) +* (0.0001∙(Xp_1_ Xp_2_∙Xp_4_)) − (0.0002∙Xp_3_^3^) + (0.0038 ∗ T^3^) − (0.04∙(Xp_2_∙Xp_4_)) − (0.11∙T^2^) − (Xp_1_^2^∙Xp_2_) + Xp_2_^3^ − Xp_2_ + (0.0002∙(Xp_3_^2^∙Xp_2_))						(10)
10	${\bar{V}}_{IER}$	0.74	0.74	Xp_1_·Xp_2_·Xp_4_ (17.04, 16.61, 0.0000) Xp_2_·Xp_4_ (−16.98, −16.94, 0.0000) Xp_1_^2^·Xp_2_ (−1.70, −10.75, 0.0000) Xp_3_^2^·Xp_2_ (7.28, 13.69, 0.0000) Xp_3_^3^ (−3.88, −12.97, 0.0000) Xp_2_^3^ (4.54, 18.48, 0.0000) Xp_2_ (−9.54, −19.07, 0.0000) H (1.28, 3.60, 0.0003) H^2^ (−1.85, −5.00, 0.0000) T^3^ (7.47, 15.95, 0.0000) T^2^ (−7.20, −14.99, 0.0000)	↑ (Xp_1_·Xp_2_·Xp_4_), Xp_2_^3^, (Xp_3_^2^·Xp_2_), H, T^3^ ↓ (Xp_1_^2^·Xp_2_), (Xp_2_·Xp_4_), Xp_3_, Xp_3_^3^, H^2^, T^2^	
Model 3. $\bar{V}=\;$ 149.66 + (Xp_1_^2^∙Xp_4_) − (1.66∙H) − (0.0001∙Xp_3_^3^) + (0.0002∙H^3^)						(11)
10	$\bar{V}$	0.28	0.28	Xp_1_^2^ Xp_4_ (0.12, 3.58, 0.0003) Xp_3_^3^ (−0.37, −11.50, 0.0000) H (−1.64, −9.72, 0.0000) H^3^ (1.29, 7.65, 0.0000)	↑ (Xp_1_^2^·Xp_4_), H^3^ ↓ Xp_3_^3^, H	

Note: Arrows (↑, ↓) indicate the direction of parameter change relative to the reference value (increase/decrease).

**Table 5:** Regression models for twill weave fabrics (uT). Dependent variables: ${\bar{V}}_{IAR}$ —intra-repeat inhomogeneity, ${\bar{V}}_{IER}$ —inter-repeat inhomogeneity, $\bar{V}$ —global inhomogeneity. Independent variables: Xt_1_—backrest roller position, Xt_2_—shed closure timing, Xt_3_—warp yarn twist direction, Xt_4_ –weft yarn twist direction.

F	Dependent Variable	R^2^	*R^2*	Most Significant Variables (Std. BETA, t, *p*-Value)	Direction of Influence (Regression coeff. B)	
Model 4. ${\bar{V}}_{IAR}=59$ .44 *+* (4.25 Xt_3_) − (0.02 H^2^)						(12)
10	${\bar{V}}_{IAR}$	0.35	0.35	Xt_3_ (0.57, 19.75, 0.0000) H^2^ (−0.14, −4.78, 0.0000)	↑ Xt_3_ ↓ H^2^	
Model 5. $\;{\bar{V}}_{IER}=\;$ 28.90 − (2.76 Xt_3_)						(13)
10	${\bar{V}}_{IER}$	0.03	0.03	Xt_3_ (−0.17, −4.74, 0.0000)	↓ Xt_3_	
Model 6. $\bar{V}=\;$ 90.56 *+* (4.87 Xt_3_)						(14)
10	$\bar{V}$	0.34	0.33	Xt_3_ (0.58, 6.95, 0.0000)	↑ Xt_3_	

Note: Arrows (↑, ↓) indicate the direction of parameter change relative to the reference value (increase/decrease).

**Table 6:** Set of weaving process parameters Xp_1_, Xp_2_, Xp_3_, Xp_4_ for three characteristic groups of plain weave fabrics (uP: P5, P12, P21), and Xt_1_, Xt_2_, Xt_3_, Xt_4_ for twill weave fabrics (uT: T9, T12, T24).

**uP**		**P5**	**P12**	**P21**	**uT**		**T9**	**T12**	**T24**
**Xp_1_**	374.4–331.2°	**374.4**	**345.6**	**360**	**Xt_1_**	100–108 cm	**102**	**106**	**104**
**Xp_2_**	95–125 cm	**102.5**	**117.5**	**110**	**Xt_2_**	360.0–303.0°	**330**	**310**	**360**
**Xp_3_**	82–90 cm	**88**	**84**	**82**	**Xt_3_**	S, S/Z, Z	**S**	**S**	**S; Z**
**Xp_4_**	5.93–31.91 cN/thread	**12.76**	**25.53**	**19.15**	**Xt_4_**	S, S/Z, Z	**Z**	**Z**	**Z**

Note: Colors indicate the three characteristic groups of fabrics analyzed: green—low inhomogeneity, yellow—medium inhomogeneity, and red—high inhomogeneity.

## Data Availability

The data presented in this study are available upon request from the corresponding author due to legal restrictions related to patent protection.

## Abbreviations

The following abbreviations are used in this manuscript:

IAR	Intra-repeat uniformity
IER	Inter-repeat uniformity
ITP	Inter-thread pore
${\bar{V}}_{IAR}$	Coefficients of the intra-repeat inhomogeneity
${\bar{V}}_{IER}$	Coefficients of the inter-repeat inhomogeneity
$\bar{V}$	Coefficients of the global inhomogeneity
Xp_1_	Shed closure timing
Xp_2_	Lease rod position
Xp_3_	Backrest roller position
Xp_4_	Pre-tension of the warp
Xt_1_	Backrest roller position
Xt_2_	Shed closure timing
Xt_3_	Warp yarn twist direction
Xt_4_	Weft yarn twist direction
