# Correction: Owczarek, M. Autoregulation of Woven Fabric Structure: Image-Based and Regression Analysis of Structural Homogeneity Under Varying Weaving Parameters. *Materials* 2025, *18*, 3554

**DOI:** 10.3390/ma19204420

**Published:** 2026-10-21

**Authors:** Magdalena Owczarek

**Affiliations:** Institute of Architecture of Textiles, Faculty of Textiles and Design, Lodz University of Technology, 90-543 Lodz, Poland; magdalena.owczarek@p.lodz.pl

In the original publication [1], there was a mistake in Equation (4). The corrected Equation (4) appears below:

(4) $${\bar{V}}_{IER}=0.2{\cdot V}_{{IER}_{A}}+0.2{\cdot V}_{{IER}_{S}}+0.2{\cdot V}_{{IER}_{P_{wa}}}+0.2{\cdot V}_{{IER}_{P_{we}}}+0.1{\cdot V}_{{IER}_{D_{ITP}}}+0.1{\cdot V}_{{IER}_{RID}}\;\;\;\;\;\;\left[\%\right]$$

There was an error regarding the affiliation. The affiliation should be updated to: Institute of Architecture of Textiles, Faculty of Textiles and Design, Lodz University of Technology, 90-543 Lodz, Poland. 

The authors state that the scientific conclusions are unaffected. This correction was approved by the Academic Editor. The original publication has also been updated.
